# Comparative Analysis of Intestinal Helminth Infections in Colic and Non-Colic Control Equine Patients

**DOI:** 10.3390/ani10101916

**Published:** 2020-10-19

**Authors:** Heidrun Gehlen, Nadine Wulke, Antonia Ertelt, Martin K. Nielsen, Simone Morelli, Donato Traversa, Roswitha Merle, Douglas Wilson, Georg von Samson-Himmelstjerna

**Affiliations:** 1 Klinik für Pferde, Allgemeine Chirurgie und Radiologie, Fachbereich Veterinärmedizin, Freie Universität Berlin, 14163 Berlin, Germany; Heidrun.Gehlen@fu-berlin.de (H.G.); nadine@netgenerator.de (N.W.); antonia.ertelt@gmx.de (A.E.); 2 M.H. Gluck Equine Research Center, Department of Veterinary Science, University of Kentucky, Lexington, KY 40546, USA; martin.nielsen@uky.edu; 3 Faculty of Veterinary Medicine, University of Teramo, 64100 Teramo, Italy; smorelli@unite.it (S.M.); dtraversa@unite.it (D.T.); 4 Institut für Veterinär-Epidemiologie und Biometrie, Fachbereich Veterinärmedizin, Freie Universität Berlin, 14163 Berlin, Germany; Roswitha.Merle@fu-berlin.de; 5 Faculty of Health Sciences, Langford Campus, Bristol Veterinary School, University of Bristol, Bristol BS40 5DU, UK; Doug.Wilson@bristol.ac.uk; 6 Institut für Parasitologie und Tropenveterinärmedizin, Fachbereich Veterinärmedizin, Freie Universität Berlin, 14163 Berlin, Germany

**Keywords:** *Strongylus vulgaris*, *Anoplocephala*, *Parascaris*, anthelmintic, cyathostomin, seroprevalence, coproscopic, helminth infection, gastro-intestinal, tapeworm

## Abstract

**Simple Summary:**

The most important equine intestinal worm species include the roundworms *Strongylus vulgaris* and *Parascaris* spp. as well as tapeworms such as *Anoplocephala perfoliata.* These parasites reside in the small and large intestine and may cause various signs of disease, such as diarrhea, unthriftiness, or colic. However, following decades of routine anti-worm treatments, it is currently unclear what relevance these infections have in the context of colic under the prevailing situation. Therefore, we examined the signs of the presence of the infection concerning the above-mentioned parasites in 620 equine clinic patients, half of these admitted after being diseased with colic and the other admitted due to non-intestinal diseases. With approximately on third of all horses being positive for antibodies directed against *S. vulgaris*, we detected an unexpectedly high infection rate. With every tenth horse showing respective antibodies, also tapeworm infections were encountered in a considerable proportion of the examined horses. Somewhat unexpectedly, no association between worm infection and colic was detected. However, recent—i.e., during the last seven days—anthelmintic treatment was 2.4 times more often seen in horses showing signs of colic. Overall, the considerable *S. vulgaris* and tapeworm infection rates mean we should stay alert in continuing worm monitoring and control.

**Abstract:**

All around the world, intestinal helminths constitute one of the most prevalent life-long occurring infections and re-infections affecting all horse age groups. A range of parasite species among strongyles, ascarids, and tapeworms is known to have the potential to cause colic in horses. However, there is a lack of current scientific evidence on the actual relevance of helminth infection levels in the context of colic in horses kept during prevailing epidemiological conditions. Thus, a prospective case-control study on the occurrence of intestinal helminths in a total of 620 mainly adult equine clinic patients was conducted to investigate the association between colic and helminth infection. For each horse, a range of copromicroscopic, serological, and clinical data was obtained, in addition to a questionnaire on relevant anamnestic data, including previous anthelmintic treatment and husbandry. Using a FLOTAC-based copromicroscopic diagnosis, the highest infection rates were seen for strongyles (41.8%), followed by *Anoplocephala perfoliata* and *Parascaris* spp. (both 0.8%), with no significant difference between the two study groups. Employing a real-time PCR a 1.1% *S. vulgaris* DNA prevalence was found. Considerably higher seroprevalences were observed using *S. vulgaris* and *A. perfoliata* ELISAs, with 32.3% and 10.7%, respectively. It was noteworthy that no association concerning either serologic status was encountered with colic status. The shedding of strongyle eggs was associated with a 1.8-times increased risk of *S. vulgaris* seropositivity. Recent anthelmintic treatment was associated with the onset of colic, as animals who had received an anthelmintic during the previous week had a 2.4-times higher risk of signs of colic compared to those who had been treated at least eight weeks prior. Another noteworthy observation was that ponies were significantly less often affected by colic than warmbloods. The high *S. vulgaris* and considerable *A. perfoliata* seroprevalences encountered in this investigation should prompt veterinarians, farm managers, and horse owners to maintain consequent and effective worm control measures.

## 1. Introduction

It is well known that infections with intestinal parasites, particularly those with helminths, can lead to serious health implications in horses, with the development of colic as one of the most important clinical signs. However, to date it is not clear to what extent and under which conditions gastro-intestinal helminths actually contribute to the etiology of currently prevailing colic cases. A complicating factor in this respect is that there are various helminth species, which differ significantly concerning their biology, pathogenicity, prevalence, and abundance. The currently most relevant helminth species in many industrialized countries concerning these aspects are the strongyles (*Strongylus* spp. and cyathostomins), ascarids (*Parascaris univalens*/*Parascaris equorum*), and tapeworms (mainly *Anoplocephala perfoliata*). Concerning these parasites, colic has been most closely associated with *Strongylus vulgaris*, *A. perfoliata*, and *Parascaris* spp. infections [1,2,3,4,5,6]. Additionally, an association between cyathostomin infections and colic is being discussed [7,8,9]; however, the evidence for this is much less solid.

For the assessment of the relative colic relevance of the above-mentioned equine helminths, it is important to be aware of their respective occurrence. According to the most recent available copromicroscopic German and international study findings, the cyathostomins to date occur almost ubiquitously amongst the strongyles and certainly at much higher rates than large strongyles such as *S. vulgaris*. The latter apparently occur only in very few horses and farms [10,11,12]. This is, however, not necessarily always the case, as recent Scandinavian studies have documented *S. vulgaris* farm prevalence of sometimes even over 60% [13,14]. Copromicroscopic data for tapeworm prevalence should always be evaluated with caution due to the very low sensitivity of the respective diagnostic procedures [15]. Nevertheless, it is well known that these parasites are also occurring in a widespread manner. Field studies have provided a wide range of coproscopic prevalences (individual horse level) from less than 5% [16,17] to 30–70% depending on the applied methodological approach [18,19]. It is noteworthy that serological testing provides sensitivities of over 80%, and thus much higher detection rates can be expected than for copromicroscopic testing [20]. However, it also has to be taken into account that antibody titers remain positive for some time, even when no parasites are present anymore—e.g., following anthelmintic treatment [21]. Nevertheless, it is thus conceivable that prevalence rates based on serological analyses are in excess of those obtained by the direct detection of tapeworms (eggs or adults), and we recently encountered this in a study involving 484 horses from 48 horse farms in Berlin/Brandenburg [22], where 16% of the tested horses from 76% of the farms were serologically positive, while *Anoplocephala* eggs were only found in 0.6% of the horses by the combined sedimentation/flotation technique [22]. Intestinal and, thus, potentially colic-causing infections with *Parascaris* spp. occur almost exclusively in foals and yearlings [3,23]. These age groups were extremely underrepresented in the present study and, thus, ascarid infections will not be addressed in this publication. Accordingly, this study focused on the occurrence of large as well as small strongyles (i.e., cyathostomins) and tapeworms, aiming to assess if any of these helminth infections were associated with colic in a cohort of approximately 300 colic patients compared with a similar number of non-colic patients. Regression analyses were performed, employing a range of parasitological and serological detection methods, together with systematic clinical examination as well as the collection of questionnaire data on aspects including horses’ clinical and treatment history. In particular, the aim was to investigate if associations between helminth infection status and colic can be detected or if other factors—e.g., previous anthelmintic treatment and husbandry—were associated with an increased risk of colic.

## 2. Materials and Methods

### 2.1. Participating Horses

All the participating horses were presented to the Equine Clinic of the Freie Universität Berlin with a history of colic (clinical signs of abdominal pain combined with reduced borborygmi, elevated heart rate, and circulatory disturbance) diagnosed by the referring veterinarian. The anamnestic diagnosis of the colic was confirmed in the patients following arrival. All the colic horses showed clinical signs of abdominal pain (scoring system, Table 1) associated with gastrointestinal disease, confirmed by further examinations. For each colic patient, a respective non-colic (i.e., orthopedic or other non-colic associated diseased) control patient of a similar age and present at the same time was assigned. A questionnaire addressing the individual details of the horse (e.g., age, breed, sex), aspects of the clinical presentation (e.g., the presence/absence of diarrhea, emaciation, recurrent colic, coughing), treatment (e.g., time point of the last anthelmintic treatment, drug used, frequency and pattern of treatment), or the farm management (e.g., number of horses, age groups, pasture size, pasture hygiene) was collected for each horse. Age-wise, the horses were categorized as foals (less than one year old), yearlings (1–3 years old), or adults (more than three years old). The following colic diagnoses were differentiated by rectal examination, surgery, and further examinations (i.e., gastroscopy): obstipation, volvulus, or incarceration of the small/large intestine; spastic colic; meteorism, strangulating colic; enteritis/colitis; equine gastric ulcer syndrome (EGUS); colic of unclear etiology; others (e.g., foreign body-associated or non-intestinal etiology such as liver or urogenital causes). All the horses underwent a general clinical examination (temperature, heart, and respiratory rate) and all the colic patients underwent a specific gastro-intestinal examination, including abdominal auscultation, rectal palpation, ultrasound examination of the abdomen, and gastroscopic examination (in cases where another colic reason could not be found). The clinical signs of colic/colic-pain were graded as no colic pain (0), low (1), moderate (2), and severe (3) (Table 1). Inclusion criteria for the colic group were the presence of clinical signs of colic pain (grade 1–3, total score >5) and a diagnosis confirming gastrointestinal disease. Exclusion criteria were no signs of colic pain or signs of abdominal pain with etiology outside the gastrointestinal tract (i.e., nephritis, myocarditis, neoplasia, cystitis, endometritis).

Conservative or surgical treatments were applied according to the respective findings and the diagnoses made based on the clinical, imaging, and surgical findings, respectively.

### 2.2. Parasitological Examinations

Fresh fecal samples (10 g) were collected from all the horses and used for macro- and microscopic analyses. The latter was performed using the FLOTAC method [24], employing saturated salt solution (specific gravity 1.2) to assess the presence as well as the quantity of helminth eggs per gram (epg) of feces. This method provides a lower detection limit of one epg. The obtained epg data were categorized as low (1–199 epg), moderate (200–499 epg), and high (>499 epg). In the case of a strongyle-positive FLOTAC examination, a larval culture using 50 g of feces was performed to generate the third stages of strongyle larvae. Larvae were frozen at −20 °C until genomic DNA was extracted from the resulting larvae using the NucleoSpin^®®^ Tissue kit (Machery-Nagel GmbH, Düren, Germany), following the mechanical disintegration of the larvae using a sterile pistil. The DNA was eluted in 50 μL of buffer and stored until further use at −20 °C.

Each DNA sample was used for the examination of the presence of nematode DNA, employing the following primers for the amplification of a 286 bp fragment of the 28S ribosomal DNA, forward primer GGCGAGTGAACGGGGAGAAGCCCAGCGCTGAA and reverse primer TTTCCTTCACAGTACTTGTTTGCTATCGAATT, following the protocol described by Demeler et al. [25]. Positive DNA samples were subsequently examined concerning the presence of *S. vulgaris* DNA employing a modification of the real-time PCR protocol established by Nielsen et al. [26]; however, instead of using a probe, SYBR green was included as a fluorescent dye in the GoTaq qPCR Master Mix Kit (Promega, Madison, WI, USA). Additionally, all the larval DNA samples were tested for the presence of 13 cyathostomin species—i.e., *Coronocyclus coronatus* (syn. *Cyathostomum coronatum*), *Coronocyclus labiatus* (syn. *Cyathostomum labiatum*), *Coronocyclus labratus* (syn. *Cyathostomum labratum*), *Cyathostomum catinatum, Cyathostomum pateratum, Cylicocyclus ashworthi*, *Cylicocyclus insigne, Cylicocyclus leptostomus, Cylicocyclus nassatus, Cylicostephanus calicatus, Cylicostephanus goldi, Cylicostephanus longibursatus,* and *Cylicostephanus minutus*—using a Reverse Line Blot (RLB) hybridization assay [27].

Blood samples were routinely taken from all the horses following admission to the clinic, and these serum subsamples were used for the analysis of the sero-prevalences of antibodies directed against *S. vulgaris* and *A. perfoliata*. An ELISA employing a recombinant protein, rSvSXP, expressed by a nematode-specific Serine-X-Proline motive-containing gene in migrating *S. vulgaris* larvae was used according to the protocol described by Andersen et al. [28], and the therein established cut-off for positive samples of 13.5% of the positive control was employed.

The detection of antibodies directed against the excretory/secretory antigens of *A. perfoliata* was performed according to the ELISA method used by Pittaway et al. [29]. Adult *A. perfoliata* were collected post mortem from the abattoir, washed in PBS, then incubated for 6 h at 37 °C in RPMI medium containing 5 μg/mL of Gentamycin. The supernatant was collected and spun at 10,000g for 15 min, filtered through a 0.22 µm filter, and the total protein content measured by a micro-fluorimeter (Quibit www.invitrogen.com). Flat-bottomed polystyrene 96-well plates (www.greinerbioone.com) were coated by overnight incubation with 100 µl/well of 0.05 M carbonate buffer at pH 9.6 containing E/S antigen at a concentration of 10 µg/mL. The antigen-coated plates were washed in PBS 0.01% tween and then blocked with PBS tween containing 1% W/V BSA. Following a further wash in PBS tween, 100 µl of the test serum diluted 1:100 with PBS tween BSA was added to each well, and a standard curve of pooled high titer serum was included on each plate. The plates were incubated at room temperature for 1 h, and following washing with goat anti equine IgG(T) horse alkaline phosphatase conjugate (AAI38AB www.bio-rad-antibodies.com), the ELISA was developed with a 1 mg/mL solution of p-nitrophenyle phosphate dissolved in 0.05 M carbonate buffer at pH 9.6; the plates were read in a dual-wavelength spectrophotometer at 405/495 nm. The results of the test samples were interpolated against the standard curve, which was assigned an arbitrary value of 100 units.

### 2.3. Statistical Analyses

Statistical analyses were performed using the program “’IBM SPSS Statistics for Windows, versiona 23 and 24 (IBM Corp., Armonk, N.Y., USA)” for OS X 10.11 and OS X 10.12.1. For the association analyses of normally distributed data (e.g., age, sex, serological and copromicroscopic data, clinical signs, and diagnoses), the χ²-Test (= Chi-Square Test) or, if that was not feasible, Fisher’s Exact Test was applied. A logistic regression model was employed, including those factors with *p* < 0.2 in χ²-Test. Continuous data were log-transformed, if necessary, to gain normal distribution. The differences between groups were evaluated by a t-Test or Mann–Whitney U-Test (in the case of non-parametric data), with *p* < 0.05 as the threshold for significance. The Kruskal–Wallis test was used to compare more than two groups, including Bonferroni corrected pairwise comparisons.

## 3. Results

### 3.1. Copromicroscopic Findings

A total of 620 horses, of which 312 were colic patients, were included in this investigation. The majority was represented by warmbloods (60%), followed by ponies (23.5%), thoroughbreds (7.8%), and cold-blooded horses (2.3%). The age of the horses ranged from 19 days to 36 years, with a median age of 11 years and 94.7% (*n* = 586) adult horses, 4.4% (*n* = 27) yearlings, and 1% (*n* = 6) foals. Almost half of the horses were geldings (49.5%, *n* = 307), 43.4% (*n* = 269) were mares, and the rest were stallions (7.1%, *n* = 44).

Of the 620 fecal samples, 263 (42.4%, 95% confidence interval (CI): 38.5–46.3%) were positive for helminth ova (representing horses from all age groups), collectively showing a broad spectrum of different parasites with strongyle (prevalence 41.9%), *Parascaris* spp. (0.8%), *Oxyuris equi* (0.2%), and *A. perfoliata* eggs (0.8%). The prevalence of helminth eggs in colic patients was 40.7% (*n* = 127) and did not differ significantly from that of the non-colic control horses with 42.9% (*n* = 132). Since the presence of helminth eggs was significantly affected by recent anthelmintic treatment, only samples of those 412 horses which did not receive a treatment during the previous eight weeks were included in the analysis of the copromicroscopic helminth prevalence (Table 1), resulting in a considerably higher overall helminth egg prevalence of 50.5% (*n* = 208, 95%-CI: 45.7–55.3%). Neither the overall prevalence of the helminth eggs nor the strongyle, ascarid, or tapeworm prevalences differed significantly between the colic and non-colic horses.

The strongyle epg counts ranged from 0 to 1282, those of *Parascaris* spp. from 0 to 41, and those of *A. perfoliata* from 0 to 27. Only one sample was positive for *O. equi*, showing an epg of 7. Strongyle eggs were found in all the age groups, whereas ascarid eggs were found only in 2–4-year-old horses and tapeworm eggs in 2–24-year-olds. Regarding the strongyle epg count, significant differences were found between adults and 1–3-year-olds, with adults having lower values (Kruskal–Wallis test, global *p* =0.011, post hoc pairwise comparison between adults and 1–3-year-olds *p* = 0.014).

The sex of the horses was not associated with the helminth egg counts (Mann–Whitney U-test, *p* = 0.364). There were no significant differences between the groups of colic and non-colic patients concerning the mean epg values of any of the helminth eggs found (*p* = 0.623). Of the 206 strongyle egg-positive horses which did not receive anthelmintic treatment during the previous eight weeks, most of the horses (80.1%, *n* = 165) were shedding low numbers and 13.6% (*n* = 28) were shedding moderate numbers of strongyle eggs, while 6.3% (*n* = 13) were shedding at least 500 epg. Amongst the latter horses, there were almost twice as many colic (*n* = 8) as non-colic (*n* = 5) patients. The fecal prevalence observed in horses which were not recently treated are shown in Table 2:

Using the Chi-square test for all the horse-related parameters, only the time point of the last anthelmintic treatment was associated with finding strongyle eggs in the facal samples (*p* < 0.001). In the multifactorial logistic regression analysis, an odds ratio of 4.2 for the risk of a strongyle-positive copromicroscopic finding was recorded for horses receiving the last anthelmintic treatment more than eight weeks prior (*p* = 0.002). Furthermore, the risk of shedding strongyle eggs was 2.8 times higher in yearlings compared with in adult horses (*p* = 0.016).

### 3.2. Serological Findings

The overall *S. vulgaris* seroprevalence (n= 609) was 32.3% (*n* = 196; 95% CI 28.5–36.4), with 31.3% in colic and 33.3% in non-colic patients and, thus, was not significantly different between the two groups. This was also true if only those horses, which did not receive an anthelmintic treatment during the previous eight weeks, were included in the analysis. A positive association was observed concerning the shedding of strongyle eggs and *S. vulgaris* seropositivity, with an OR of 1.4 (*p* = 0.035), which increased to 1.7 (*p* = 0.02) if only those horses which were not treated with an anthelmintic during the previous eight weeks were included.

A total of 619 samples were tested for *A. perfoliata* IgG(T) antibodies, providing an overall seroprevalence of 10.7% (*n* = 66; 95% CI 8.4–13.1), with no significant difference between the group of colic and non-colic patients. Amongst the five horses with *A. perfoliata* eggs detected in their feces, four were found to be seropositive. Additionally, with 559 ELISA units, the mean *A. perfoliata* antibody titers were significantly higher in the horses shedding tapeworm eggs than in the negative horses with only 13 units (Mann–Whitney U-test, *p* = 0.006).

### 3.3. Molecular Findings

The DNA of a total of 177 larval samples was used for the amplification of *S. vulgaris* ribosomal DNA, providing seven positive results (4%), of which three samples were derived from colic patients. Accordingly, the overall *S. vulgaris* DNA prevalence, if calculated for all the study horses, would be 1.1%, and no significant difference between colic and non-colic patients was encountered.

**Figure 1:**
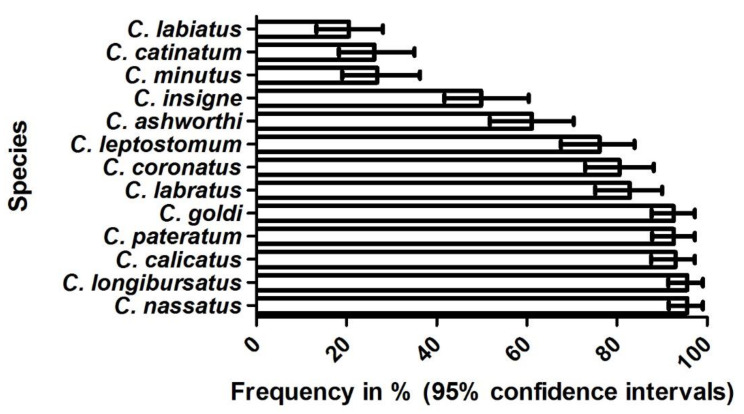
Frequency of 13 cyathostomin species detected by Reverse Line Blot analysis of DNA samples extracted from the cultured larvae obtained from horses which did not receive anthelmintic treatment during the prior eight weeks and showed strongyle eggs in their feces (*n* = 195). Bars indicate the occurrence in %, and 95% confidence intervals are provided as Ⱶ.

Using the DNA extracted from those 206 horses with a strongyle egg-positive fecal sample and the last anthelmintic treatment received at least eight weeks prior, 195 valid RLB results were obtained. Overall, for all 13 cyathostomin species included in the diagnostic spectrum of the test, positive results were obtained; however, there were major species differences concerning the prevalence (Figure 1). Only 58.5% of all samples provided a positive RLB result, whereas if only the samples with a strongyle epg of at least 50 were included, 94.8% were RLB-positive for at least one species. No significant association was seen between the outcome of the RLB test and the colic status of the horses.

### 3.4. Clinical Findings

Concerning the individual horse criteria, only for breed was a correlation with the colic status observed, suggesting that ponies had an OR of 0.66 (*p* = 0.032) compared with warmbloods for developing colic symptoms.

An overview of the clinical signs potentially associated with parasite infection and exhibited by colic patients prior to admission according to their owners was provided for 82 (26.3%) of the colic patients (Table 3). For patients without colic, the owners reported generally lower percentages of clinical signs. Concerning the clinical signs, an increased risk of having a positive *Parascaris* spp. sample was only recorded for horses with diarrhea; however, as indicated above, this was based on only five positive samples.

**Figure 2:**
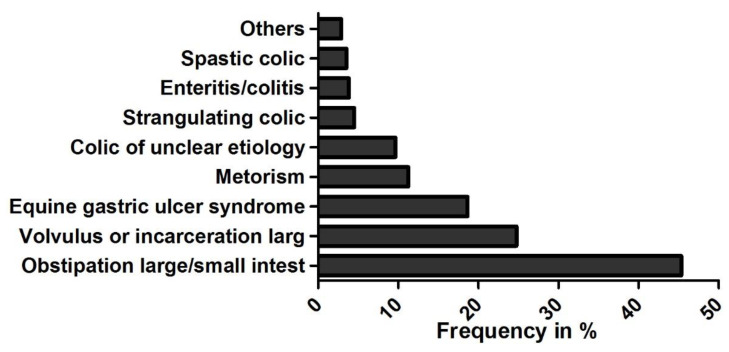
Colic diagnoses following admission % (*n* = 311).

For nearly half of the colic patients (45.3%) an impaction of the small or large intestine (most commonly an impaction of the ascending colon) was diagnosed following admission. In 24.8%, an intestinal displacement was recorded (Figure 2).

In 18.6% of the colic patients, the reason was regarded to be a primary overload of the stomach, and in 9.3% ulceration of the stomach mucosa was diagnosed.

No statistical significant dependence was recorded between any of the colic diagnoses established following admission and the copromicroscopic or serological strongyle findings (*p* = 0.771). Noteworthy, colic patients with an obstipation of the ascending or descending colon had a 17 times higher risk of a positive copromicroscopic tapeworm result, which, however, was just not statistically significant (*p* = 0.053).

The chance of a positive strongyle egg finding was increased 4.4 times (95%-CI 1.8–10.9) in horses which had received their last anthelmintic treatment at least eight weeks prior compared to those who were treated within the week prior (Chi-squared test, *p* < 0.001). For 268 horses, information on the anthelmintic product/compound used for the most recent treatment was recorded, and in 77.2% (*n* = 207) of the cases a product containing a macrocyclic lactone (ivermectin 184, moxidectin 23; both with and without praziquantel), in 15.7% (*n* = 42) a product containing pyrantel, and in 5.2% of the cases a benzimidazole (*n* = 14) was used. None of the colic patients having received anthelmintic treatment one week prior to admission was treated with a benzimidazole. Interestingly, the risk of horses exhibiting signs of colic was 2.4 times higher in horses who had received their last anthelmintic treatment during the last week prior to admission (*n* = 22), compared to those who had received their last treatment at least eight weeks prior (*n* = 171; *p* = 0.025).

## 4. Discussion

This study aimed at a systematic investigation of potential associations between the occurrence of clinical signs of colic/severity of colic pain and the direct or indirect evidence of gastro-intestinal helminth infections in horses. To this end, both a clinical colic evaluation as well as an extended set of parasitological examinations was performed on the two study groups, each composed of approximately 300 colic and non-colic patients of the equine clinic at the Freie Universität Berlin, Germany. For most of the obtained prevalence data, similar findings were reported during previous studies in Germany [12,30,31,32,33,34,35,36], particularly if one takes into account the age structure of the horses within the present study, which was dominated by adult horses, with only few yearling and hardly any foals. Accordingly, of those horses which did not receive a recent anthelmintic treatment, strongyle egg shedding was seen in approximately every second horse, and even if recent treatment was not taken into account, still more than 40% were strongyle-positive. Using molecular techniques, a similar relative representation of the species belonging to the examined spectrum of small strongyle species has been encountered in an earlier German study [12], with the species *C. nassatus, C. longibursatus, C. pateratum*, and *C. goldi* found to occur in over 85% of the samples and *C. labiatus* as well as *C. minutus* belonging to the least often detected species in both studies.

In contrast to previous studies reporting a less than 2% S. vulgaris prevalence in Germany using larval culture or DNA-based copromicroscopic methods [10,16], the high *S. vulgaris* seroprevalence of 32.3% was unexpected and is also astonishing in view of the herein calculated overall fecal *S. vulgaris* DNA prevalence of 1.1%. However, this is actually less than the 62.2% reported in the only other European field investigation of *S. vulgaris* sero-prevalence published recently by Scandinavian colleagues [36], who, in a very similar approach, compared gastro-intestinal parasite infection with intestinal disease status in a total of 259 horses. In that study, the fecal *S. vulgaris* DNA prevalence (5.5%) was also considerably higher than obtained for the patients in the Berlin clinic, mirroring the higher seroprevalence data. Noteworthy, neither our data nor that of the latter Swedish study obtained any statistical association between colic and *S. vulgaris* positivity. This is in agreement with previous investigations on the occurrence of *S. vulgaris* [37] and also cyathostomins [38,39] in colic versus control horses. However, there are some publications describing the opposite, such as *S. vulgaris*-associated colic due to non-strangulating intestinal infarction [3]. Nevertheless, it appears that *S. vulgaris* infections may be rather associated with peritonitis as according to Pihl et al. [4], who observed increased white blood cell counts, total protein, and lactate levels in the peritoneal fluid associated with this parasite infection, and the majority did not present with colic. Additionally, Tyden et al. [14] encountered higher *S. vulgaris* antibody titers in horses with peritonitis. Since, in the present study, the peritoneal fluid was not routinely examined, it is not possible to comment on this association.

Regional differences in the occurrence of *S. vulgaris* have been hypothesized to be associated with the frequency of selective versus strategic anthelmintic treatment control [13]. Due to the six months prepatent period of this parasite, it is hypothesized that the life cycle can be effectively disrupted with a minimum of two yearly treatments. However, if, as recommended for selective treatment, only horses exceeding a certain strongyle fecal egg output receive anthelmintic treatment, some horses may not receive any treatment for more than a year [40]. As a matter of fact, recent studies have shown that, in countries with an increased use of selective treatment approaches such as Sweden and Denmark, the *S. vulgaris* prevalence increased upon the widespread application of this strategy [13,14]. This is in agreement with recent investigations on the occurrence of cyathostomins [38] or *S. vulgaris* [37] in colic versus control horses.

The effect of anthelmintic treatment on the strongyle population was documented by the observation that horses receiving treatments less than eight weeks prior were less prone to shedding strongyle eggs. Similar observations were made by Hedberg-Alm et al. [37], who reported that if treatment was more than three months prior, the risk of higher strongyle epg counts were significantly higher.

The herein obtained low copromicroscopic prevalence of *A. perfoliata* of 1% confirms the most recent study data [22] obtained during field investigations performed in the same geographical region (i.e., Berlin/Brandenburg), where in 0.6% of the examined 481 samples cestode eggs were detected. Approximately 10 years earlier, Hinney et al. [16] recorded a prevalence of 14.3% when examining 1407 equine fecal samples from 126 Brandenburg farms. It remains unclear why this major reduction in copromicroscopic tapeworm prevalence occurred, as neither study was epidemiologically representative and aspects such as anthelmintic treatment intensity or frequency were not comparatively assessed. However, the observed 10.7% *A. perfoliata* seroprevalence indicates that the parasite is considerably more common than copromicroscopic data suggest. This is corroborated by a sero-epidemiological study conducted most recently using both a serum and a saliva-based *A. perfoliata* ELISA, in which prevalences of 16.2% and 29.5% were recorded, respectively, on nearly 50 Berlin/Brandenburg horse farms [22]. As these two studies are the first serological tapeworm investigations in Germany, it is not possible to assess any trends across time. In line with previous investigations reporting associations between copromicroscopic and serological *A. perfoliata* infection status [41], our findings suggest a higher risk for a sero-positive *A. perfoliata* result in horses shedding tapeworm eggs. However, it has to be taken into account that we encountered only five horses shedding tapeworm eggs in their feces and, thus, this observation is not based on sufficient data to allow any meaningful statistical analysis. The low number of horses with tapeworm eggs detected in their feces must be seen in context with the sensitivity of the employed copromicroscopic method. Nielsen [3] summarized studies on the sensitivity of several copromicroscopic techniques for the detection of *A. perfoliata* eggs describing sensitivities of less than 50% for all flotation methods not employing centrifugation steps. To the best of the authors’ knowledge, no evaluation on the sensitivity of the FLOTAC method for the detection of *Anoplocephala* eggs has been published. However, Rinaldi et al. [42] reported that this method and using saturated salt as flotation solution provided superior results in terms of precision and accuracy concerning the analysis of *Monezia* spp. eggs in sheep feces compared with simple flotation and McMaster protocols.

Generally, the anamnestically reported clinical signs were not associated with the detection of parasite infections, even though a considerable proportion of the colic patients had previously suffered from signs often related to parasite infections, such as recurrent colic or wasting.

We did not see any association between tapeworm infection status/antibody titer and colic. This lack of association between tapeworm infection status and colic was similarly observed by a Swedish study [41], although other studies—e.g., [1,2,43]—have reported associations between tapeworms and ileocecal colic. The reasons for this discrepancy remain unclear and may, for example, be associated with the intensity of infection and/or anthelmintic treatments in the respective horses but may also be due to the rather broad definition of colic employed in this study (see below).

No association was encountered of copromicroscopic or serological findings with the most common diagnoses (impaction of the large/small intestine and small intestinal displacement).

In this context, it is relevant to highlight that based on recent studies it becomes apparent that worm infections in horses seem to rather be associated with specific clinical conditions, such as non-strangulating infarction in *S. vulgaris* infections, which is only rarely present as colic cases [3], or ileal/ileocecal impactions in tapeworm infections (for a review, see Nielsen et al. [15]). Accordingly, due to the broad colic definition employed in the present study, it can be assumed that such *S. vulgaris* or tapeworm-associated colic types did not become apparent.

A noteworthy observation was that horses, which had received an anthelmintic treatment (only MLs, pyrantel and praziquantel) during the week prior to hospitalization, had a significant, i.e., 2.4 times, higher risk for colic than patients receiving anthelmintic treatment a longer time ago. A similar observation was made by Barret et al. [44], who described the onset of colic and diarrhea within 12 h post treatment with a combination of ivermectin and praziquantel in horses seropositive for *A. perfoliata*. Another study also reported on the potential for the development of colic signs within 24 h post anthelmintic treatment, but this only in *Parascaris* infected horses with an age of up to 12 months where prior treatment with either MLs or pyrantel led to small intestinal obstruction through the present ascarids [6]. Thus, this situation is neither concerning the age of the horses nor the colic type comparable with that of the horses in the present study. It remains unclear due to which reason recent anthelmintic treatment appeared to be associated with an increased risk for colic, and this finding should stimulate further investigations of this matter.

To conclude, it appears that under the prevailing conditions of helminth infections, the examined cohort of horses did not suffer from gastro-intestinal helminths infection intensities resulting in sufficient damage to significantly contribute to the development of colic signs. However, our findings confirm earlier observations and provide several important insights, e.g. the lack or at least the very limited clinical colic relevance of patent cyathostomin infections was confirmed. It is also noteworthy that based on our findings, the prevalence of *S. vulgaris*, for which presence was herein documented by serological examination, has to be considered to be much higher than currently assumed based on copromicroscopic studies. Additionally, we observed a lower risk for colic in ponies compared with warmbloods, and a potential association with recent anthelmintic treatment using neurotoxic compounds and colic. Furthermore, a strikingly higher tapeworm sero- compared to copromicroscopic prevalence indicates that these parasites may occur much more commonly than expected based on fecal analysis.

## 5. Conclusions

Overall, the findings from this study suggest that, under the prevailing husbandry and treatment conditions, intestinal helminths played a minor role as causes of colic in the cohort of clinic-admitted equines.

## Figures and Tables

**Table 1:** Grading of the clinical signs of horses with colic.

Parameter	Symptoms	Grading
Bowel peristalsis	Normal motility.	0
	Reduced motility.	1
	No motility.	2
	Hypermotility.	3
Kicking to the stomach	Horse stands still, no kicking to the stomach.	0
	Occasionally kick against the stomach (1–2 times in 5 min).	1
	Kicks regularly against the stomach (3–4 times in 5 min).	2
	Kicks excessively against the stomach (>5 times in 5 min).	3
Pawing	Horse stands still, no pawing.	0
	Occasional pawing (1–2 times in 5 min).	1
	Regular pawing (3–4 times in 5 min).	2
	Excessive pawing (>5 times in 5 min).	3
Head movements	No sign of discomfort, head is mainly held straight in front of the body.	0
	Intermittent, lateral, or vertical head movements, occasionally looking at the flank (1–2 times in 5 min) and/or lifting the lips (1–2 times in 5 min).	1
	Intermittent, violent, lateral, or vertical head movements, looking regularly at the flank (3–4 times in 5 min) and/or lifting the lips (3–4 times in 5 min).	2
	Continuous head movements, looking excessively at the flank (>5 times in 5 min) and/or lifting the lips (>5 times in 5 min).	3
Lying down, rolling	Horse stands quietly in the box.	0
	Occasionally laying down.	1
	Regularly lying down and getting up again, rolling.	2
	Horse repeatedly throws itself down uncontrollably and rolls on the ground.	3

**Table 2:** Prevalences of eggs from gastro-intestinal helminths in horses which did not receive anthelmintic treatment during the previous eight weeks (*n* = 412).

	Prevalence (%) [95% Confidence Interval]		
Parasite(s)	Total	Colic patients	Non-colic controls
Helminths	50.5 [45.9; 55.2]	49.5 [41.8; 56.3]	51.4 [45.1; 57.6]
Strongyles	50.2 [45.7; 54.9]	49.5 [41.6; 56.3]	50.9 [44.7; 57.2]
*A. perfoliata*	1 [0.2; 2]	0.5 [0; 1.6]	1.4 [0; 3.3]
*Parascaris* spp.	0.5 [0; 1.2]	0 [0; 0]	0.9 [0; 2.3]

**Table 3:** Distribution of the clinical signs potentially associated with parasite infection reported in colic patients signs prior to admission (colic *n* = 82; control *n* = 306).

	**Percent of Colic Patients with Clinical Signs Listed Below**				
Last deworming	Coughing	Diarrhea	Emaciation	Recurrent colic	Reduced performance
Irrespective (*n* = 82)	12.2	8.5	22	68.3	4.9
≥8 weeks (*n* = 53)	9.4	9.4	17	77.4	3.8
	**Percent of Control Patients with Clinical Signs Listed Below**				
Last deworming	Coughing	Diarrhea	Emaciation	Recurrent colic	Reduced performance
Irrespective (*n* = 306)	3.3	2.3	5.9	18.3	1.3
≥8 weeks (*n* = 196)	2.6	4.5	4.6	35.9	2.6
